# Enhancing inguinal tumor treatment outcomes with a customized 3D‐printed bolus

**DOI:** 10.1002/acm2.70175

**Published:** 2025-07-14

**Authors:** Xing Li, Stephen Bhagroo, Fan‐Chi Su, Robabeh Rahimi, Jonathan Tward

**Affiliations:** ^1^ Department of Advanced Radiation Oncology and Proton Therapy Inova Schar Cancer Institute Fairfax Virginia USA; ^2^ Department of Radiation Oncology University of Utah Salt Lake City Utah USA; ^3^ Department of Radiation Oncology University of Texas Southwestern Medical Center Dallas Texas USA; ^4^ Department of Radiation Oncology University of Maryland School of Medicine Baltimore Maryland USA

**Keywords:** 3D‐Printing, patient‐specific treatment device

## Abstract

Traditional boluses for pelvic lesions present challenges, including compromised skin dose coverage from large air gaps in treatment plans and increased uncertainties in treatment delivery due to variations in bolus fitting. This study evaluates the clinical benefits of a patient‐specific, 3D‐printed bolus (pBolus) in external beam radiation therapy (EBRT) for extramammary Paget's disease in the inguinal fold. A three‐dimensional (3D) conformal plan utilizing the pBolus was compared to a conventional Superflab bolus (sBolus) under identical beam arrangements. The pBolus improved dose conformity by reducing air gaps in the treatment plan, and therefore, it was selected for clinical treatment. Weekly setup imaging revealed air gaps predominantly along the inguinal fold, demonstrating overall consistency in bolus fitting with minor air gap variations. Additionally, in vivo dosimetry confirmed dose accuracy. As a result, the patient achieved a favorable outcome, with no clinical evidence of disease at the follow‐up visit.

## 1 INTRODUCTION

Three‐dimensional (3D) printing technology, valued for its precision and cost‐effectiveness, is increasingly utilized in radiation oncology for quality assurance (QA) and the fabrication of treatment accessories,^1^, ^2^ including playing a pivotal role in customizing patient‐specific boluses in photon^3^ and electron^4^ external beam radiation therapy (EBRT) to improve tumor dose coverage and, ultimately, treatment efficacy.

A conventional Superflab bolus (Eckert & Ziegler, Massachusetts, USA) conforms well to flat skin surfaces like the chest wall, offering effective treatment outcomes for postmastectomy breast radiation.^5^, ^6^ However, when applied to curved anatomical areas, such as the ear, nose, or scalp, the soft material of Superflab tends to create substantial air gaps and inconsistent interfractional bolus placement, which potentially increase dosimetric uncertainties in treatment plans and treatment delivery. In contrast, a 3D‐printed bolus (pBolus), directly derived from patient computed tomography (CT) images, conforms more precisely to complex anatomy, reducing air gaps and providing dosimetric advantages, as demonstrated in both phantom and patient studies.^7^, ^8^, ^9^ Additionally, the bolus's rigid or semi‐rigid material ensures more consistent bolus placement across treatments.

The 3D‐printing workflow generates a virtual bolus structure from the patient's CT images, then edits and slices it into layers of print‐ready files.^10^ Subsequently, the bolus is printed with various thermoplastic polymers, such as acrylonitrile butadiene styrene (ABS), polylactic acid (PLA), or thermoplastic polyurethane (TPU), using an inexpensive fused deposition modeling (FDM) printer, or with resin using a high‐end stereolithography (SLA) printer.^11^ Print quality is determined by material selection, nozzle speed, in‐fill patterns, and staff experience.^12^, ^13^, ^14^ Post‐processing is often required, either through mechanical smoothing or chemical treatments, depending on the material and printer type. As a result, QA of the printed bolus is essential to ensure the absence of internal defects such as air gaps. Overall, the turnaround time for a customized bolus, from design to final product, can range from hours to days. Therefore, implementing 3D‐printing for bolus in a radiation oncology clinic is resource‐intensive, requiring specialized skills and staff training.^15^ It should only be considered for lesions where conventional bolus methods are inadequate.

Pelvic tumors are a prime example that could benefit from a pBolus.^16^ They are often located in highly curved anatomical areas, where Superflab tends to create large air gaps and inconsistent bolus fitting. In contrast, the advantages of using a pBolus for pelvic tumors were demonstrated in a prior phantom study,^17^ paving the way for its application in clinical cases.

This study presents a pBolus designed for inguinal tumor treatment in EBRT, aiming to demonstrate its dosimetric benefits and improved placement reproducibility compared to the conventional Superflab bolus (sBolus).

## 2 METHODS

**FIGURE 1:**
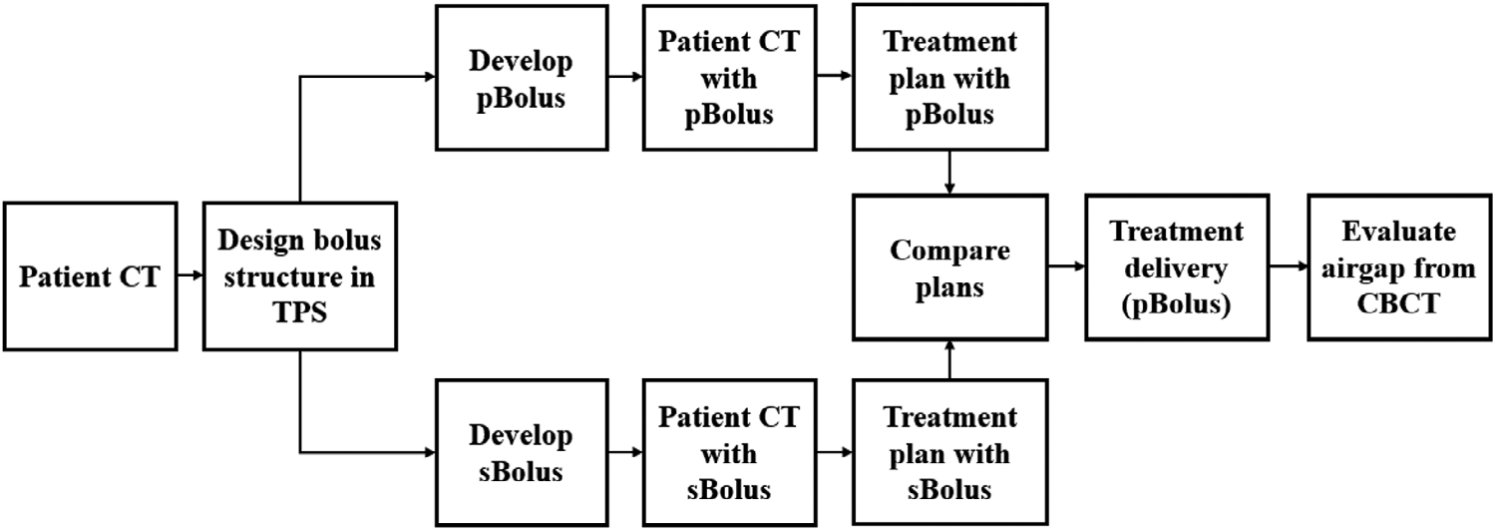
Study workflow using a pBolus and an sBolus. pBolus, 3D‐printed bolus; sBolus, Superflab bolus.

A 75‐year‐old patient with a history of melanoma and extramammary Paget's disease in the left inguinal fold received 60 Gy in 30 fractions using a 3D‐conformal technique with 6 MV photon beams in EBRT. Two treatment plans were created for comparison: one with a conventional sBolus and another with a pBolus. Air gaps were assessed using weekly cone beam CT (CBCT) setup images. Figure 1 illustrates the study workflow.

### 2.1 Bolus design

The patient was imaged using a pelvic CT protocol (120 kV, 2.5 mm slice thickness). In the Eclipse treatment planning system (Varian Medical Systems, Palo Alto, California, USA), the planning target volume (PTV) and surrounding organs‐at‐risk (OARs) were delineated. Based on the CT images, a virtual bolus structure with a uniform 8 mm thickness was designed to ensure adequate dose coverage to the PTV. The virtual bolus was refined in 3DBolus software (Adaptiiv Medical Technologies Inc., Halifax, Canada) and exported to slicing software for 3D printing.

**FIGURE 2:**
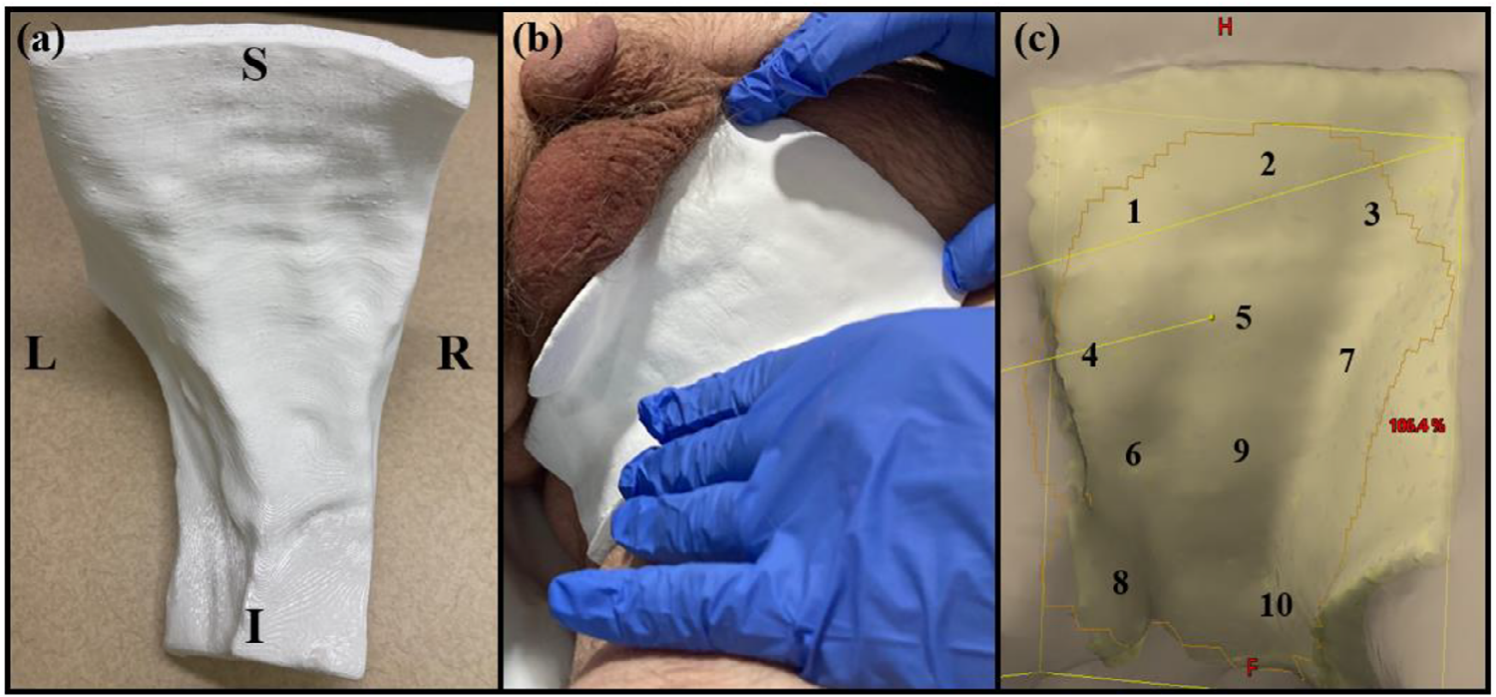
(a) The 3D‐printed bolus. S, I, L, and R refer to superior‐inferior and left‐right orientations relative to the patient. (b) Bolus placement on patient. (c) In vivo dose verification. Dosimeters were placed under the bolus at locations labeled 1 to 10.

An FDA‐approved PLA material (1.24 g/cc) was selected and printed directly using an FDM printer (Raise3D Pro2 Plus) with the slicing software Raise3D IdeaMaker (Raise3D Technologies, Irvine, California, USA). The printed bolus was smoothed with a Dremel tool to enhance patient comfort without altering its thickness. A CT scan of the pBolus was performed as a QA measure to verify structural integrity and ensure the absence of internal defects, such as air gaps, per the American Association of Physicists in Medicine (AAPM) Task Group 199 (TG‐199) report.^18^ Figure 2 illustrates the pBolus and its placement on the patient. A 10 mm thick sBolus (1.00 g/cc) was also created for plan comparison. The radiological equivalent path lengths of the two boluses were designed to be identical.

### 2.2 Treatment planning and delivery

**FIGURE 3:**
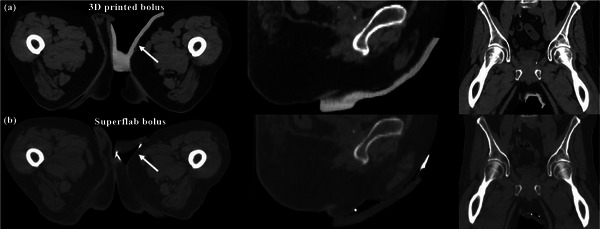
CT images of (a) a 3D‐printed bolus and (b) a Superflab bolus for the same patient. From left to right: axial, sagittal, and coronal views. CT, computed tomography.

The patient was imaged with both the sBolus and pBolus for treatment planning, as shown in Figure 3. The two treatment plans had identical beam arrangements, using 6 MV photon beams and a 3D conformal technique for EBRT. Dose calculation was performed with the Acuros 16.1 algorithm in the Eclipse treatment planning system (TPS). The air gaps were manually contoured in each axial CT slice using consistent Hounsfield units (HU) thresholds.

To guide bolus selection, air gap volumes and dose distributions were compared between the pBolus and sBolus plans. Marked skin locations were used to ensure consistent bolus placement during setup. To assess variations in air gap volumes, weekly CBCT setup images were retrospectively registered to the planning CT. Air gaps were also manually contoured in the CBCT using consistent HU settings. Throughout the treatment, a total of 10 optically stimulated luminescent dosimeters (OSLDs) were placed between the bolus and the skin surface. To minimize potential air gaps introduced by the dosimeters, two OSLDs were used per fraction, covering the entire treatment area as shown in Figure 2c. The in vivo measured doses were then compared with the estimated doses from the TPS.

## 3 RESULTS

**FIGURE 4:**
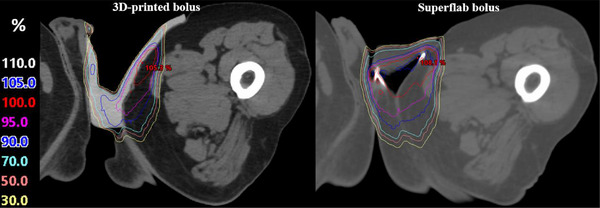
Dose distribution in the (left) pBolus plan and (right) sBolus plan. pBolus, 3D‐printed bolus; sBolus, Superflab bolus.

**FIGURE 5:**
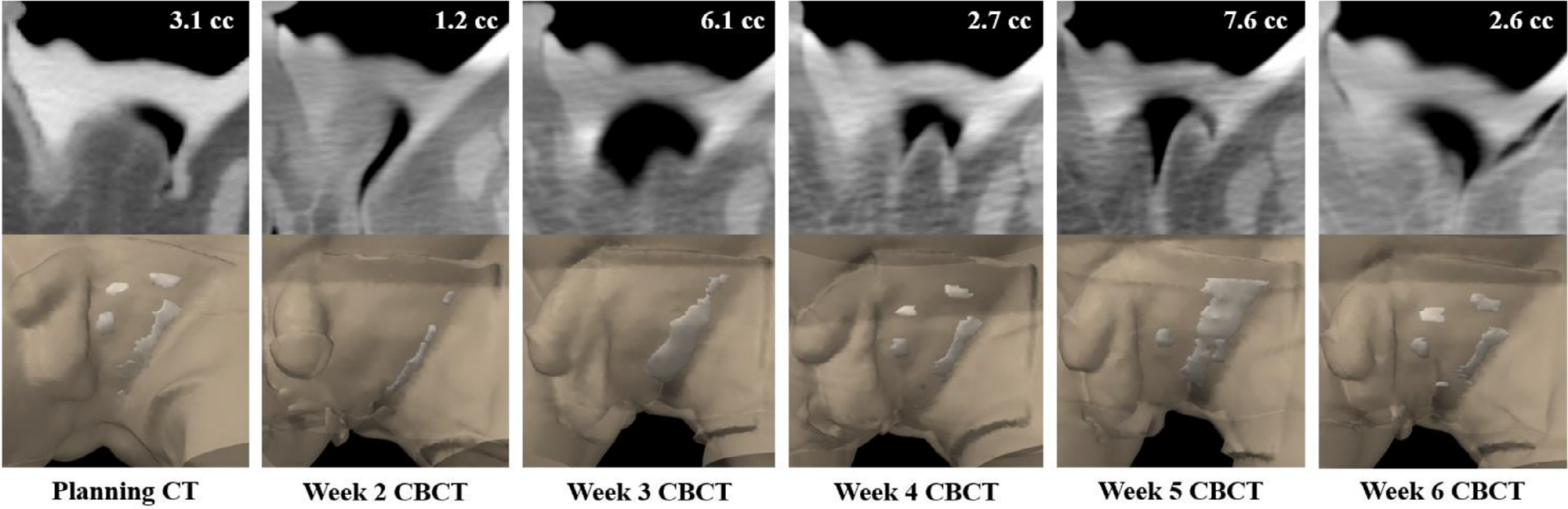
Weekly variation in air gap volume.

**FIGURE 6:**
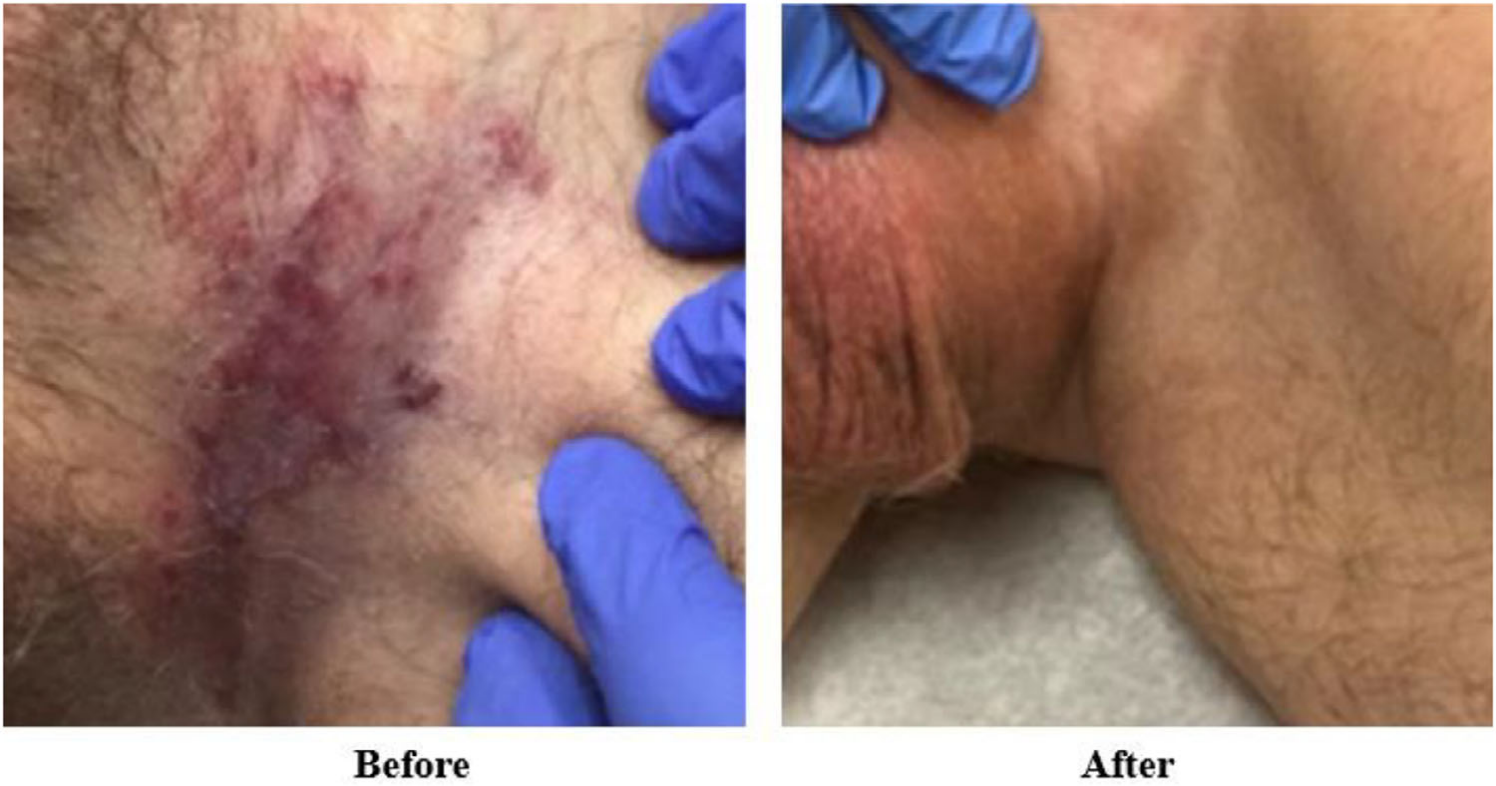
Comparison of before versus after EBRT treatment. EBRT, external beam radiation therapy.

As shown in Figure 3, the pBolus plan exhibited a significantly smaller air gap volume (3.1 cc) compared to the sBolus plan (12.5 cc) and achieved a more conformal dose distribution, as demonstrated in Figure 4, leading to its selection as the clinical plan. Table 1 quantifies the dosimetric metrics according to the Radiation Therapy Oncology Group (RTOG) 0630 Trial,^19^ showing a 3.1% increase in PTV coverage with the pBolus plan, a 7% reduction in rectal dose, and up to a 27.2% reduction in testis dose. The OSLD‐measured doses closely matched the TPS‐predicted doses, with the maximum percentage of differences +4.7% occurring near the inguinal fold. Dose discrepancies at other locations remained within ±5%. Figure 5 illustrates the weekly air gap variations. Consistent with the treatment plan, most air gaps appeared near the inguinal fold area during treatment delivery, and the patient tolerated the rigid PLA bolus well. At the 3‐month follow‐up visit, radiation oncologists examined the patient's groin area and found no evidence of Paget's disease, as shown in Figure 6.

**TABLE 1:** A comparison of dosimetric metrics from the pBolus and sBolus plans.

Target/OAR	DVH^a^	Treatment plan	
		sBolus^b^	pBolus^c^
PTV	V95%	96.0%	99.1%
Rectum	V30Gy	23.9%	16.9%
Testis (left)	V45Gy	100%	98.5%
	V50Gy	98.4%	91.9%
	V60Gy	33.0%	5.80%
Bladder	Dmax	4.00 Gy	3.40 Gy
Femur (left)	Dmax	29.8 Gy	29.6 Gy

^a^ DVH = Dose volume histogram,

^b^ sBolus = Superflab bolus,

^c^ pBolus = 3D‐printed bolus.

Abbreviations: OAR, organs‐at‐risk; PTV, planning target volume.

## 4 DISCUSSION

The patient did not respond well to topical treatment, a common therapy for Paget's disease, making EBRT the only viable option. Compared to a conventional sBolus, the pBolus reduced the air gap volume, leading to a more conformal dose distribution and improved PTV coverage. Bolus placement remained reproducible, consistently maintaining low air gap volume during treatment. As a result, the patient achieved a positive clinical outcome, with no evidence of Paget's disease observed at the 3‐month follow‐up visit after EBRT.

Bolus fitting significantly influenced treatment efficacy. Table 1 shows that the air gaps resulted in smaller dose differences between the pBolus and sBolus in PTV coverage compared to OARs sparing at deeper depths, aligning with previous studies.^20^, ^21^ Variations in air gaps along the inguinal fold, as illustrated in Figure 5, highlight the importance of consistent skin markings and experienced personnel in bolus placement to minimize interfractional variability. This observation is supported by findings from Malone et al.,^22^ who reported changes in the fit of a pBolus for vulvar lesions over the course of treatment.

Direct PLA printing was selected for its numerous benefits. First, PLA is an FDA‐approved, biodegradable polymer, making it safe for patient use.^22^, ^23^ With a tissue‐equivalent density of 1.24 g/cc, it produces less scattered dose from photoneutrons,^24^ a factor not accounted for by the Eclipse TPS. Second, the direct printing workflow is less time consuming than the mold‐casting method,^8^ making it more suitable for our busy clinical setting. Third, the PLA's rigidity is beneficial for treating lesions near the inguinal fold, where soft tissues make reproducibility challenging. While FDM printing offers a cost‐effective solution, its lower resolution compared to SLA printing results in increased air gaps, necessitating re‐CT imaging to account for dosimetric discrepancies in treatment plans.^22^

It is critical to understand that print quality depends on materials and printer settings. Craft et al. characterized the variance in HU across different printable materials, emphasizing the need to evaluate a material before its use in patient care.^12^ Biltekin et al. later reinforced this by showing that even with the same material, factors such as infill pattern and printing direction affected the dosimetric properties of the printed bolus.^13^ The evidence underscores the necessity for QA of the pBolus in radiation therapy to ensure precise and safe treatment for patients. Recently, Adaptiiv Medical Technology (Halifax, Canada) launched an FDA 510(k) cleared service to customize patient‐specific, 3D‐printed treatment accessories in EBRT and Brachytherapy, including a workflow of QA for the 3D‐printed device as a standardization of care. Additionally, AAPM has formed Task Group No. 336 to address the need for QA for 3D printing in radiation therapy applications.

This study has limitations. First, the fabrication of the bolus requires several hours, potentially extending treatment planning turnaround times and limiting feasibility in high‐throughput clinical settings. Second, OSLDs have an inherent dose measurement uncertainty of ±5%, as outlined by the AAPM Task Group 191 report,^25^ which may impact the precision of dose verification results.

## 5 CONCLUSION

We introduce an effective treatment option using a 3D‐printed bolus in EBRT for Paget's disease in the inguinal fold. Compared to conventional boluses, the reduction in air gaps with the printed bolus enhanced dose conformity and increased the percentage of target volume receiving at least 95% of the prescription dose by 3.1%. Additionally, the printed bolus exhibited consistent interfractional fitting during treatment delivery. Consequently, the patient achieved a positive clinical outcome, with no clinical evidence of Paget's disease at the follow‐up visit.

## AUTHOR CONTRIBUTIONS

XL designed the 3D‐printed bolus, developed the study workflow, and drafted the manuscript. SB performed in‐vivo dosimetry. FS contributed to bolus design and manuscript revisions. RR provided feedback on the initial draft and subsequent revisions. JDT contributed to study design and writing. All authors reviewed and approved the final manuscript.

## CONFLICT OF INTEREST STATEMENT

The authors declare no conflict of interest related to this study.
